# Proactive postgraduate education in disaster medicine and preparedness for enhanced disaster management

**DOI:** 10.1186/s12909-026-08638-5

**Published:** 2026-02-14

**Authors:** Jonas Zimmerman, Amir Khorram-Manesh, Yohan Robinson, Diana Swolin-Eide, Viktor Glantz, Eric Carlström, Joakim Björås

**Affiliations:** 1 Institute of Clinical Sciences, Sahlgrenska Academy, University of Gothenburg, Gothenburg, Sweden; 2 Center for Disaster Medicine, University of Gothenburg, Gothenburg, Sweden; 3 Gothenburg Emergency Medicine Research Group (GEMREG), Sahlgrenska Academy, Gothenburg University, Gothenburg, Sweden; 4 Department of Pediatrics, Institute for Clinical Sciences, Sahlgrenska Academy, University of Gothenburg, Gothenburg, Sweden; 5 Region Västra Götaland, Department of Research, Development, Education and Innovation, Sahlgrenska University Hospital, Sahlgrenska University Hospital, Gothenburg, Sweden; 6 Region Västra Götaland, Department of Pediatrics, Region Västra Götaland, Department of Research, Development, Education and Innovation, Sahlgrenska University Hospital, Queen Silvia Children’s Hospital, Sahlgrenska University Hospital, Sahlgrenska University Hospital, Gothenburg, Sweden

**Keywords:** Disaster medicine management, Disaster education, Interprofessional collaboration, Simulation-based training, Crisis leadership, Health system preparedness

## Abstract

**Introduction:**

Disaster preparedness is crucial for managing complex emergencies, yet educational frameworks are often underdeveloped or inconsistently applied across national contexts. This study introduces and evaluates the PRAD-MED (Preparedness and Disaster Medicine) program, a novel educational initiative developed in Sweden to enhance disaster response among healthcare professionals. Inspired by global competency frameworks, the program aimed to integrate theoretical, practical, and collaborative learning approaches, nationally and internationally, to strengthen organizational and individual preparedness in a structured manner.

**Methodology:**

The study employed a mixed-methods approach. Quantitative data were collected through a 29-question survey, assessing participants’ knowledge, confidence, and perceived relevance of the program. Focus group interviews and observational assessments provided qualitative insights into experiences and the programs impact on interdisciplinary collaboration, leadership, and organizational preparedness. The program featured a modular design with tabletop and modular simulation exercises.

**Results:**

Sixteen physicians participated, representing diverse medical specialties. Quantitative findings showed significant improvements in knowledge, with 94% of participants rating their post program disaster medicine proficiency as “high” or “very high.” Leadership and collaboration skills were highly rated, with 87% identifying enhanced decision-making and teamwork capabilities. Qualitative data, including observers reports emphasized the transformative impact of simulations and cross-sectoral exercises, fostering a deeper understanding of roles and communication in disaster scenarios.

**Conclusion:**

The PRAD-MED program demonstrated strong potential to address existing gaps in disaster medicine education by equipping healthcare professionals with the knowledge and skills required to serve as future instructors in disaster simulation and preparedness. By emphasizing leadership, interdisciplinary collaboration, and practical exercises, the program likely enhanced participants’ readiness for future emergencies. Ongoing participant involvement in subsequent course rounds is believed to have fostered a sustainable and expanding training model. Continued development through broader stakeholder engagement and integration of advanced training technologies may further strengthen long-term disaster preparedness across Sweden.

**Supplementary Information:**

The online version contains supplementary material available at 10.1186/s12909-026-08638-5.

## Introduction

Disaster management education has evolved globally in response to rising disaster risks. As in many countries, disaster medicine is not formally recognized as a medical subspecialty in Sweden. Access to academic postgraduate training in disaster medicine is still limited to a few higher educational centers [1]. This has led to knowledge gaps in medical disaster management and a lack of skills among responders and managers to address public health and disaster risk scenarios [2]. Furthermore, there is a lack of global standardization in defining core competencies for disaster medicine and management [3, 4]. The American Medical Association’s Center for Public Health Preparedness and Disaster Response developed a consensus-based educational framework and competency set to improve disaster medicine and public health preparedness [5]. This framework includes seven core learning domains, 19 core competencies, and 73 specific competencies for healthcare personnel. The goal was to standardize disaster response training for healthcare professionals, address preparedness gaps, and equip them with the necessary strategic and tactical abilities. This competency-based approach emphasizes practical skills and a multidisciplinary perspective, ensuring consistent disaster medicine education in healthcare settings [5]. Although there are several competency-based curricula in disaster medicine, most of the countries have either adopted or modified the American curriculum [6].

Based on this, in 2015, a standardized European disaster management curriculum was proposed to address inconsistencies in training across countries, emphasizing the need for harmonized education for medical professionals, emergency personnel, and policymakers [2]. The curriculum covered risk assessment, preparedness, response, recovery, and resilience, with training tailored to different roles and incorporating multidisciplinary perspectives. E-learning, regular updates, and international collaboration were recommended to share resources and best practices [2]. Furthermore, a global curriculum with seven core competency domains, including strategic and tactical skills for collaboration was suggested. The curriculum would feature modular courses for various managerial levels, fostering continuous professional growth and potentially evolving into a year-long master’s program [7].

Although the suggestions were not fully implemented, academic programs increasingly include disaster and emergency management in their educational portfolio, with initiatives like The Federal Emergency Management Agency’s (FEMA) Disaster Management University focusing on competency-based training for resilience [8]. Advanced technologies such as augmented reality (AR), virtual reality (VR), and artificial intelligence (AI), are revolutionizing preparedness through immersive simulations, particularly in developing nations and private sectors [9].

In Europe, frameworks like the European Qualification Framework ensure standardized competencies for professionals [10]. Community and youth engagement efforts, often led by organizations like FEMA, and similar European centers emphasize grassroots training for localized resilience [8, 11]. However, challenges persist, including limited funding, lack of awareness, and technology adoption barriers, especially in low-resource regions. Nevertheless, the current geopolitical tension and post-COVID experiences have opened up new opportunities to improve preparedness and response efficiency in crises among healthcare employees.

### Developing the program structure

In 2021, Sahlgrenska University Hospital in Gothenburg, Sweden, formed a group of emergency and disaster medicine specialists, and global health experts, to develop a postgraduate preparedness and disaster medicine training program. Initially tailored for physicians, the program was created to enhance the hospital’s preparedness by incorporating lessons from the recent pandemic and addressing an increasingly uncertain geopolitical landscape [12].

Having extensive experience in designing educational programs, the task was assigned to the Department of Research, Education, Development, and Innovation. Initially targeting residency physicians, the program also attracted specialist physicians, ensuring broad representation across medical fields. A project manager structured the program and recruited suitable participants. Two physicians with field experience were recruited as course directors. Throughout the developing process, a reference group including pedagogic and subject-matter expertise provided feedback to ensure quality and comprehensiveness.

### Purposes and goals

The PRAD-MED (PReparedness And Disaster MEDicine) program aimed to develop sustainable competence among hospital physicians in disaster management, preparedness organization, and leadership through education and practical exercises. The goal was to enhance the hospital’s preparedness capabilities and ensure that knowledge was applied across departments. A key objective was to foster networking among participants and internal and external stakeholders for improved collaboration, creating a foundation for initiating improvements across sectors after the completion of the program. Additionally, the program sought to establish a qualified simulation platform for continuing collaborations and regular exercises, strengthening preparedness and emergency response capabilities.

### Program structure and content

The PRAD-MED program was constructed to combine theoretical lectures, instructor-led workshops, and interactive exercises, focusing on organization and leadership rather than clinical skills [2, 7]. Participants were set to visit key actors within the Swedish total defense sector (civil-military coordination and integration of civil society) and engage in experience-sharing with national and international experts. The program prioritized local and regional experts, but also included guest lecturers from national and international organizations. Authorities such as the Swedish Civil Defence and Resilience Agency, the Swedish Armed Forces, the National Board of Health and Welfare, law enforcement, emergency services, and various universities actively contributed to the program.

The program consisted of seven weeks of full-time education across three modules. Modules one and two were three weeks long, while module three was one week, focusing on final exercises and training. The modules were spaced approximately 4 to 6 weeks apart, allowing participants to continue their clinical duties while gradually building knowledge and skills. Between active course weeks the participants worked on assignments assessing and improving preparedness efforts within their departments, in alignment with the hospital’s overall disaster plan. The program commenced in September 2023 and concluded in September 2024, ensuring a balance between professional development and ongoing clinical responsibilities.

### Module 1 - disaster medicine organization, leadership, and governance (3 weeks)

The first module aimed to provide a foundational understanding of disaster medicine organization and leadership at global, national, and local levels, covering governance and decision-making processes in healthcare during disasters, conflicts, and in peacetime. Special focus was placed on the healthcare’s role within the Swedish total defense system and the national and regional crisis preparedness organizations. The module was intended to explore the relationship between international and national disaster response plans. Content included global health perspectives across low-, middle-, and high-income settings, international humanitarian law, human rights, and key bilateral and multilateral conventions. Participants’ also received training in leadership and the incident command system (ICS), with a focus on defined roles and responsibilities within the response organization. The module further provided comparative opportunities to analyze the Swedish disaster management framework in relation to international models and practices. Additionally, tabletop exercises and related activities were introduced.

### Module 2 - disaster medicine knowledge, skills, and training (3 weeks)

The second module focused on applying medical leadership competencies and disaster medicine in practice. Participants were trained to manage and lead operations in complex and high-risk environments along with other key components, including disaster triage and the principles of initial management during disasters. One prominent feature was further development of the tabletop simulation exercises that tested the ability to make effective decisions under pressure, while participants’ were continuously assessed by experienced instructors. This aimed to improve individual competence and coordination between stakeholders in disaster preparedness and offered an opportunity for course leaders to assess each individual’s performance. The module also introduced a modular simulation system using patient cards as markers.

### Module 3 - disaster medicine practice, training, and collaboration

The final module combined competence from the previous modules with practical application. Interactive lectures were complemented by hands-on activities aimed at enhancing skills in managing and governing disasters and emergencies. This module emphasized collaboration with key stakeholders in societal preparedness, culminating in a large-scale collaborative exercise. Participants were also trained as regional instructors in the introduced patient card simulation system, enabling them to apply their skills in realistic disaster simulations (Table 1).

**Table 1:** Shows the key topics covered during the course. CSCATTT stands for: Command and Control, Safety, Communication, Assessment, Triage, Treatment, Transport. 3LC stands for: Three-Level Collaboration.

Module	Main Theme	Key Topics Covered
Module 1: Disaster Medicine Organization, Leadership, and Governance	Foundations and policy	Swedish total defence; Crisis organization (local, regional, national); Global humanitarian systems; International law; Civil-military coordination; incident command system; leadership roles in crises; Introduction to simulation exercises, Basic disaster medicine and management in resource-limited settings.
Module 2: Disaster Medicine Knowledge, Skills, and Training	Tactical application	Disaster triage principles; Medical leadership under pressure; Decision-making under uncertainty; Tabletop simulations; Modular training with MAss Casualty SIMulation (MACSIM); The use of CSCATTT as collaborative factors.
Module 3: Disaster Medicine Practice, Training, and Collaboration	Collaborative exercises	Large-scale disaster scenario; Stakeholder coordination; 3LC exercises; Cross-sectoral planning; Role-specific preparedness; MACSIM instructor certification

### Collaborative factors and simulation exercises

Several simulation systems have been used in tabletop and modular exercises. However, a few have been tested with collaborative factors. Recent publications have demonstrated the successful integration of such factors into exercises for both collaborative training and skill evaluation. This includes the CSCATTT framework; Command and Control, Safety, Communication, Assessment, Triage, Treatment, and Transport - from the Major Incident Medical Management and Support (MIMMS) courses. These elements have also been incorporated into the Three-Level Collaboration (3LC) tabletop exercises and the more advanced modular simulation training, Medical Response to Major Incidents (MRMI) [13–16].

The 3LC model is a validated tabletop exercise model that involves participants from operational, tactical, and strategic levels working together in simulated disaster scenarios. At the operational level, frontline staff handle the direct response. The tactical level involves mid-level managers coordinating resources and operations. At the strategic level, senior leaders make high-level decisions and provide policy support. These exercises test communication and coordination across all levels to improve overall disaster response and identify any gaps in the system [13, 15].

Medical Response to Major Incidents (MRMI) is a training program designed to equip healthcare professionals with the necessary skills and knowledge to respond to large-scale emergencies effectively. Using patient cards from the MAss Casualty SIMulation system (MACSIM), derived from real events, the MRMI course typically covers topics like triage, mass casualty management, incident command, coordination with emergency services, and resource management under pressure. This aims to prepare participants to handle the complexities of major incidents, ensuring that they can provide efficient, life-saving care while working within a broader response system [12–14, 16]. Collaboration, shared mental models, and situational awareness can be facilitated through the use of the CSCATTT framework, which aims to transform theoretical knowledge into practical performance during simulations. This allows participants to experiment with different strategies and make mistakes in a safe environment, leading to enhanced learning without any risk of harm [17].

### Aim

This study aimed to evaluate the outcome of a new disaster medicine and preparedness program (PRAD-MED), inspired by earlier studies encompassing a modular structure, and collaborative exercises with multiprofessional participants.

## Methods and materials

### Study participants

Sixteen physicians were recruited to participate in the study. Inclusion criteria were participation in the entire PRAD-MED training program, age between 18 and 69 years, and current employment as a resident or specialist physician at Sahlgrenska University Hospital. Participants who did not complete the full program were excluded.

### Study methods and rationale for a combined approach

To ensure a comprehensive evaluation of the PRAD-MED program, a multi-layered mixed-methods approach was employed, integrating quantitative, qualitative, and observational components. The design allowed triangulation of data sources, combining standardized quantitative instruments with qualitative insights to capture individual learning outcomes and broader organizational effects.

Several practical measures were implemented to maintain methodological consistency and manage resource demands. Observational and focus group components were supported by standardized rubrics and digital tools to reduce scoring variability and facilitate data integration. Self- and peer-assessments were collected anonymously to minimize bias, and observers underwent calibration prior to data collection. Representative sampling and virtual follow-up sessions reduced logistical demands.

Participants’ who completed all program modules were awarded instructor-candidate certification, requiring continued participation in subsequent courses and simulations. This structure validated acquired competencies and promoted long-term retention through sustained engagement in the training and simulation cycles. The multi-layered evaluation framework is summarized in Table 2.

**Table 2:** Evaluation framework of the PRAD-MED program

Evaluation Layer	Tool/Framework	Purpose	Type of Data	Strengths
Self-Assessment	29-item Likert questionnaire (pre/post) [18]	Measure perceived competence and confidence	Quantitative	Standardized, assessed by external experts
Observational Assessment	CSCATTT-anchored instructor scoring during exercises [19]	Evaluate applied performance in disaster domains	Quantitative/ Behavioral	Domain-specific, allows targeted feedback, validated in 3LC context
Peer Feedback	Post-session structured debriefs [20]	Promote reflection and peer learning	Qualitative	Adds social learning dimension
Focus Group	Semi-structured interview [21]	Explore perceived learning, context, and organizational effects	Qualitative	Deep insight, triangulates quantitative data

### Quantitative study based on a questionnaire

#### Preparing the questionnaire

The course directors, physicians with academic and field experience in disaster management jointly developed a 29-item questionnaire based on the CSCATTT framework [13–15]. This ensured balanced coverage of all major thematic areas addressed during the program. Participants’ completed the questionnaire on the final day of the PRAD-MED program.

The questions encompassed all key components of disaster and emergency management defined by the CSCATTT model: organization, leadership, safety and security, assessment, communication, triage and prioritization, treatment, logistics, and transportation. Before inclusion, items underwent multiple rounds of review, editing, and refinement to ensure clarity and relevance. Validation procedures are described below (Table 3).

**Table 3:** Survey questions used for the quantitative evaluation

Survey questions
How would you rate your knowledge in disaster medicine prior to the program?
How would you rate your knowledge in disaster medicine after the program?
How would you evaluate your ability to perform in a disaster scenario in your regular role?
Was the program’s overall structure well-organized and effective?
Was the program length well-suited and appropriately balanced?
The program content was engaging and of high quality?
It is beneficial to start with a global perspective and conclude with a local perspective?
It was beneficial to combine lectures with practical exercises?
I have the opportunity to utilize my new knowledge in my practice.
I have learned about leadership and its function in a disaster medical event.
My knowledge of collaboration between actors in a disaster medical event has increased during the program.
I have gained knowledge about important actors outside of the healthcare sector in disaster medical events?
I have learned about the importance of efficient coordination between actors during a disaster medical event
I have learned about safety and security considerations during a disaster medical event
I have learned about the importance of effective communication in a disaster medical event
I have learned about the importance of a shared assessment in a disaster medical event.
I have learned about triage during disaster medical events
I have gained a deeper understanding of medical transport during disasters and its significance
The 3 Level Collaboration exercises were an efficient way to enhance teamwork and decision-making skills
The MACSIM cards were useful for deepening my understanding of triage and decision-making in a disaster medical event
Modular exercises with MACSIM are an effective way to gain insight into disaster medical management
The following lectures have been particularly valuable to me
The following topics and components were missing from the program
How well does the program align with the stated objectives?
What significance do you perceive the program has for your continued work in the field of disaster medicine?
How would you assess the level of the information provided during the program?
What is your overall assessment of the program?
Did the program meet your expectations as before enrolment?
To what extent has your executive chief been involved in your activities and progress during the program?

#### Validation of the questionnaire

Three independent reviewers, two senior experts in course design and one junior researcher, assessed the instrument’s validity according to standard criteria [22]:

- *Face Validity*: Ensured the questionnaire measured the intended constructs.
- *Construct Validity*: Ensured the items accurately reflected the theoretical concepts being evaluated.
- *Content Validity*: Ensured the questionnaire covered all relevant areas of disaster medicine education.
- *Criterion Validity*: Ensured the responses corresponded to expected outcomes of the educational intervention.

#### Knowledge assessment

Participant learning and performance were evaluated using a combination of self-assessment, structured reflection, and instructor observation, designed to capture both perceived and demonstrated competence development.

The primary quantitative measure of individual improvement relied on self-assessment. In disaster training, self-assessment is a recognized and valuable method for promoting reflection, identifying strengths and weaknesses, and supporting readiness for future response [23]. To complement this, recurring supervision and structured reflection were incorporated throughout the course. Instructors provided continuous one-on-one feedback, and each training day began with short reflective sessions focusing on the previous day’s activities, achievements, and areas for improvement.

To evaluate the participants’ self-perceived knowledge, a validated instrument adapted from the previously mentioned 3LC exercise framework was employed. This model, widely applied in disaster and emergency management education emphasizes: (1) understanding of cross-agency roles; (2) inter-agency communication and information flow; and (3) joint problem-solving and decision-making [24–26].

These levels closely mirrored the pedagogical objectives of the PRAD-MED program with a focus on fostering collaboration, situational awareness, and the development of shared mental models in complex, multi-agency environments. The assessment process incorporated three complementary components: (1) Post-scenario seminars: structured discussions in which participants’ identified performance gaps, analyzed shortcomings, and proposed targeted improvements; (2) Participant feedback: surveys used to evaluate perceived usefulness, learning outcomes, and progress in collaboration and leadership; and (3) Analysis of decision-making: systematic examination of how participants and agencies jointly addressed problems and made operational decisions during simulated scenarios, providing insight into collaborative effectiveness.

In addition to these self- and peer-evaluative components, formal observational assessment was conducted by experienced instructors using CSCATTT-anchored scoring to evaluate participants’ applied performance during simulations. Continuous observations were systematically discussed among lead instructors to ensure calibration and capture individual progress over time. This structured, domain-specific approach complemented the self-assessment data and provided longitudinal insight into participants’ behavioral competence and practical performance in disaster medicine.

### Qualitative interview

#### Design

A focus group interview was conducted to explore experiences and perceived learning outcomes. Focus groups encourage participant interaction and collective reflection, making them particularly suitable for evaluating complex educational programs [27]. Although the ideal group size is typically 8–12 participants, a single group of 16 was used to ensure consistent interpretation across the cohort. This approach allowed for efficient evaluation of teamwork, leadership, communication, and problem-solving, skills central to the program’s objectives.

Based on the CSCATTT framework used in the questionnaire, a semi-structured interview guide was developed to examine views on course content, learning needs, and suggestions for improvement [12–15]. The guide was piloted and refined before use. Open-ended questions encouraged detailed responses, while structured moderation ensured adherence to research objectives. Experienced interviewers mitigated common drawbacks of large focus groups, such as dominance by outspoken participants or reduced input from introverted individuals, through targeted facilitation and balanced participation [23, 28].

#### Procedure

The focus group interview was conducted on the final day of the PRAD-MED program and lasted approximately 40 min. Audio recordings and field notes were collected in a quiet setting following participant consent. Recordings were transcribed verbatim, proofread against the audio, and timestamped to ensure accuracy. All data were anonymized in accordance with confidentiality and ethical guidelines [29].

An inductive content analysis was applied to address the study objectives [27]. Statements were coded into meaning units, grouped into subcategories and categories, and synthesized into overarching themes. Representative quotations were selected based on thematic recurrence and relevance, following established qualitative reporting standards.

### Ethical approval

This study adhered to the ethical guidelines outlined in the Declaration of Helsinki and the Swedish Ethical Review Act. Informed consent was obtained from all participants to take part in the study, assuring they could withdraw at any time without facing any negative consequences. The study was approved by the Swedish ethical review authority (no 2024-07483-01).

## Results

### Demographics and participant overview

The PRAD-MED program included 17 medical doctors. Sixteen of the 17 enrolled (94%) completed both the questionnaire and the interview and were included in the study. Table 4 summarizes the demographic characteristics of the participants, including gender, age, level of training, and medical specialty. A majority were resident physicians, and the representation of specialties was diverse. Of note, only a minority had previous deployment experience or prior involvement in preparedness and disaster medicine planning activities.

**Table 4:** Demographics of the participants in the study

Demographics	*n*
Participants	16
Gender	
Male	7
Female	9
Age y (mean)	40
Residents	10
Specialists	6
Emergency medicine	3
Anesthesiology	4
Internal medicine	2
Surgery	3
Psychiatry	2
Radiology	1
Ophthalmology	1
Experience of disaster medicine	
Deployed to a disaster	3
Not deployed to a disaster	13

### Quantitative results of the questionnaire

#### Knowledge improvement

**Fig. 1:**
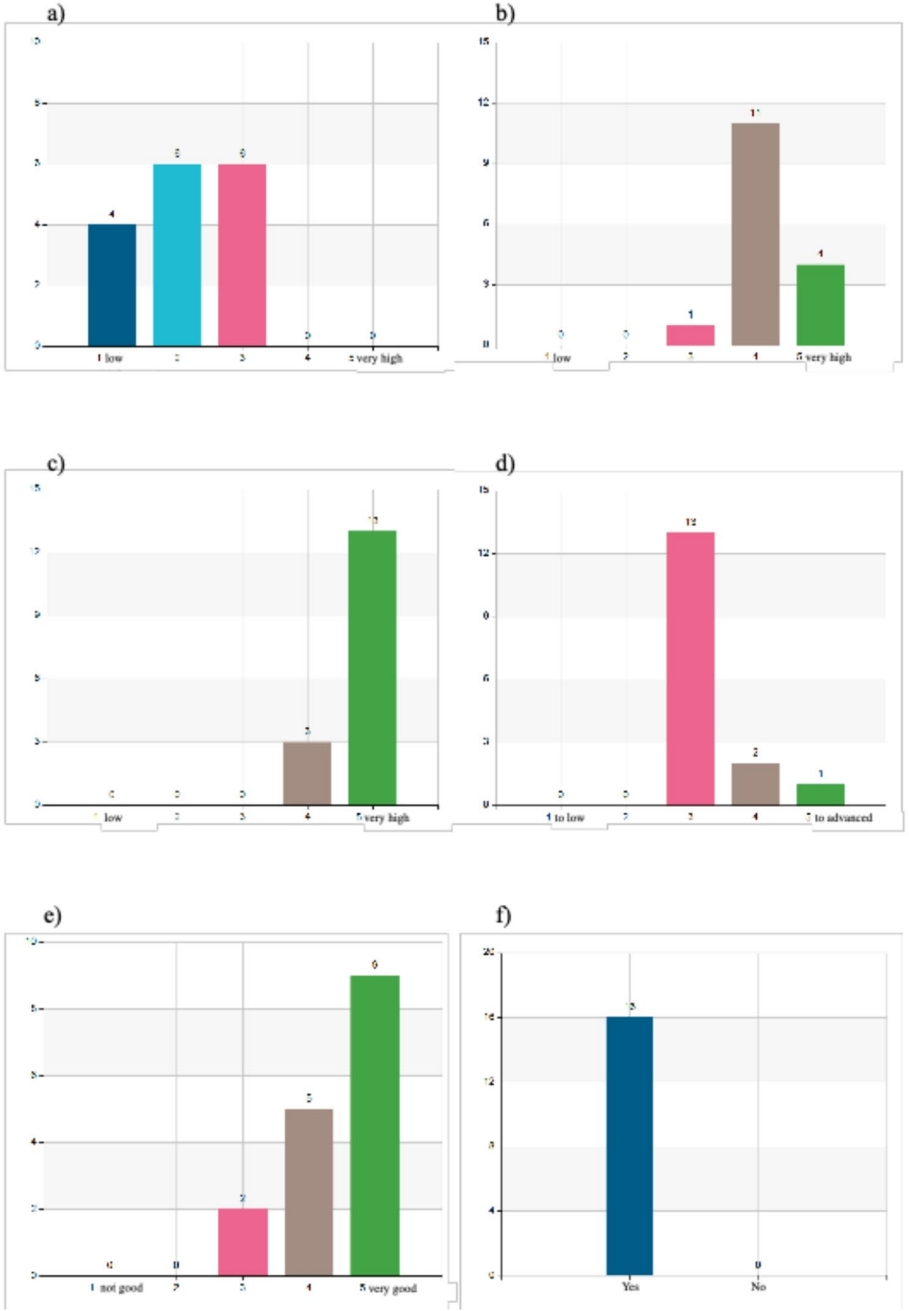
**a** How would you rate your knowledge in disaster medicine prior to the course? **b** How would you rate your knowledge in disaster medicine after the course? **(c)** What significance do you perceive the course have for your continued work in disaster medicine? **d** How would you assess the level of the information provided during the course? **e** What is your overall assessment of the course? **f** Did the course meet your expectations as prior to enrolment?

A 5-point Likert scale (1 representing “low” and 5 representing “very high”) was used for the quantitative questionnaire on self-perceived knowledge improvement. Participants reported low to moderate prior knowledge in disaster medicine, with 75% rating themselves at level 2 or 3 and 25% at level 1 (Fig. 1a).

Following the program, self-assessed knowledge increased markedly, with 94% rating themselves at level 4 or 5 (Fig. 1b). The overall experience was positively received: 87% rated the program as 4 or 5, and all participants agreed that it met their expectations (Figs. 1e–f). In terms of relevance for future professional practice, 81% rated it as highly relevant (5), with the remaining 19% rating it as 4 (Fig. 1c). Most participants (81%) also found the level of information well balanced (Fig. 1d). Full response distributions are presented in Fig. 1.

#### Content of the program

**Fig. 2:**
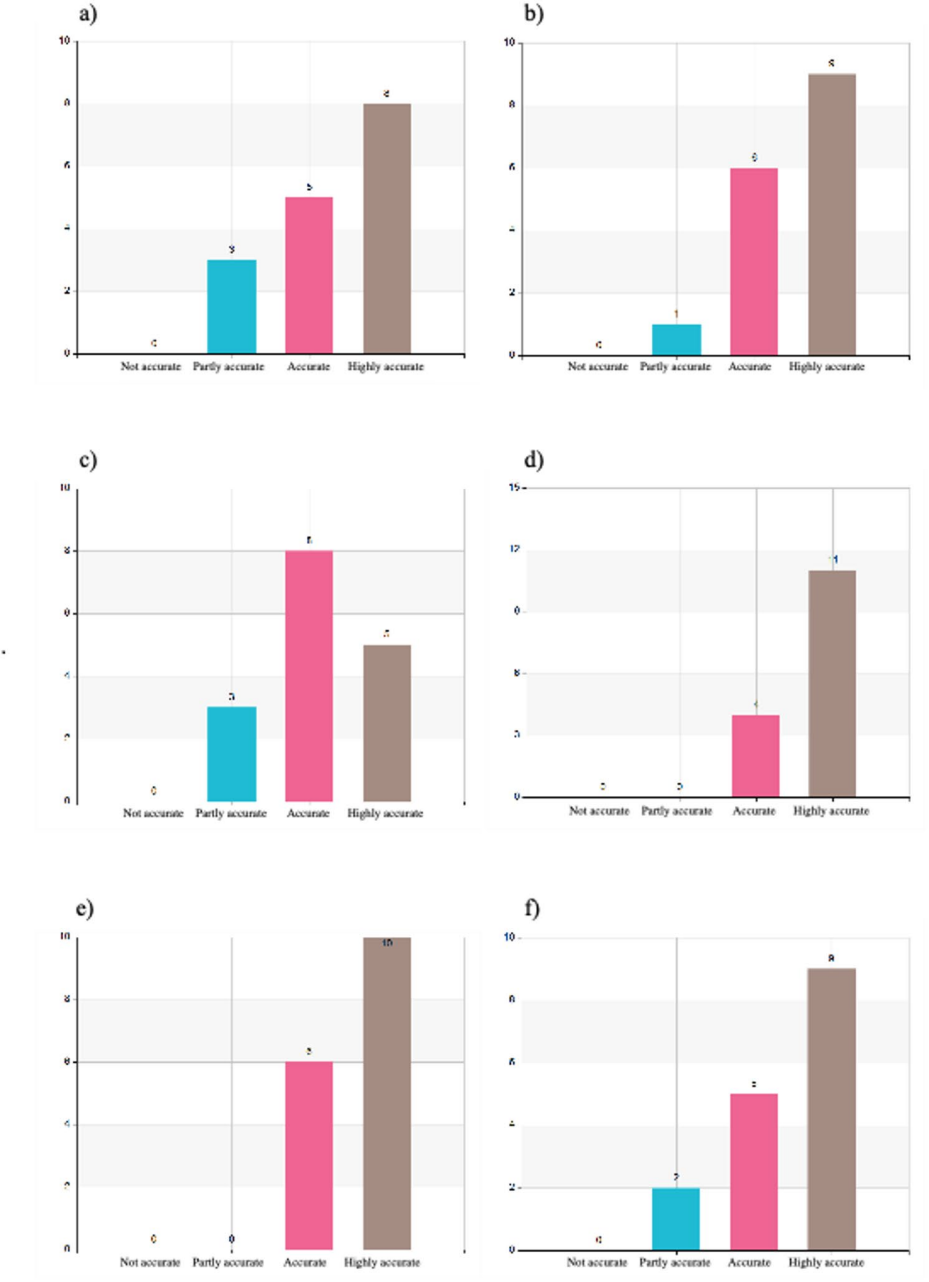
**a** I have the opportunity to utilize my new knowledge in my practice. **b** I have learned about leadership and its function in a disaster medical event. **c** I have learned about safety and security considerations during a disaster medical event. **d** I have learned about the importance of effective communications in a disaster medical event **(e)** I have learned about the importance of efficient coordination between actors during a disaster medical event. **f** My knowledge of collaboration between actors has increased during the course

**Fig. 3:**
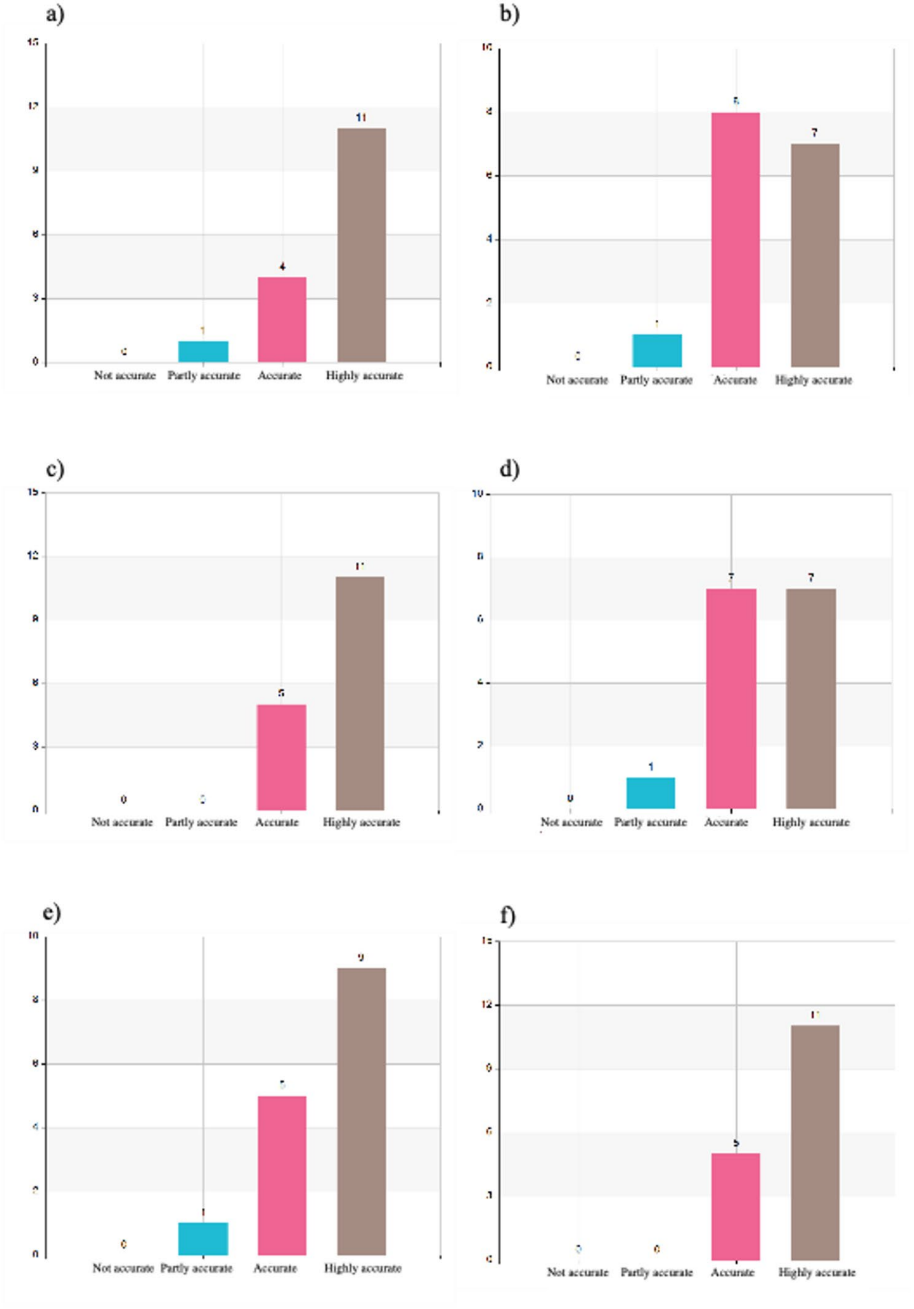
**a** I have gained knowledge about important actors outside of the healthcare sector in a disaster medical event. **b** I have learned about the importance of a shared assessment in disaster medical events. **c** I have learned more about triage during disaster medical events. **d** The MACSIM cards were useful for deepening my understanding of triage and decision-making in a disaster medical event. **e** Modular exercises with MACSIM are an effective way to gain insight into disaster medical management. **f** It was beneficial to combine lectures with practical exercises

The content of the program was rated using a 4-point scale ranging from “not accurate” to “highly accurate.” Participants consistently rated the program content positively across multiple domains. The balance between theoretical and practical components was unanimously appreciated, with 69% rating it as “highly accurate” and 31% as “accurate” (Fig. 3f). Relevance to everyday clinical practice was also affirmed, with 50% rating this aspect as “highly accurate” and 31% as “accurate” (Fig. 2a).

Leadership and command training - a recognized gap in traditional medical education - was well received, with 56% rating it as “highly accurate” and 38% as “accurate,” (Fig. 2b). Communication and coordination were particularly strong components: 73% rated their understanding of communication in disaster settings as “highly accurate” (Fig. 2d), while 63% gave the highest rating to insights into intersectoral collaboration (Fig. 2e).

The program also strengthened understanding of the roles of non-health actors such as government and military agencies, rated as “highly accurate” by 69% (Fig. 3a). Shared situational assessments were positively received, with 44% giving the highest rating (Fig. 3b). Practical tools such as disaster triage and the MACSIM simulation were well evaluated, with 69% and 60% respectively rating them as “highly accurate” (Fig. 3c and e).

Full distributions of participant responses across all content areas are provided in Figs. 2 and 3.

### Qualitative results of the interviews

The findings from the survey were reinforced during plenary interviews, where participants elaborated on the transformative nature of the program from their professional perspectives.

#### Impact on leadership

The program prompted reflection on the differences between routine hospital operations and the rapid decision-making required during crises. Participants highlighted the tension between maintaining everyday organizational structures and adapting them to disaster scenarios. One participant commented as follows:

*"There is a stark difference between leading in a routine linear structure and during a crisis. Leading within the framework of the former works well under usual circumstances, but it becomes a bottleneck during extraordinary events. The current system is not structured for minute-to-minute operations, which makes rapid adaptation difficult without prior planning and understanding of crisis-specific workflows. To lead during crisis demands a more agile approach, this is something that our system does not naturally accommodate." *

This realization underscores the need for leaders to develop a dual skillset - one balancing day-to-day management and the other to build the agility needed in disaster scenarios. Additionally, participants’ emphasized the value of the MACSIM simulation exercises in preparing for leadership roles in disaster management. The exercises provided hands-on experience in managing mass-casualty incidents and building individual confidence and team cohesion. One participant commented:

*“The MACSIM exercises taught me the importance of understanding the balance between guiding a team effectively and adapting quickly to evolving situations. They reinforced the need for a leader to coordinate seamlessly with different specialties while maintaining focus on the bigger picture”​*.

#### Enhanced understanding of interdisciplinary and cross-sectoral collaboration

A major focus of the program was fostering collaboration between stakeholders in disaster management, including healthcare professionals, emergency services, and government agencies. Participants’ reported significant learning in this area, highlighting an increased understanding of how to get engaged with prehospital ambulance services, law enforcement, and other critical actors. One participant remarked:

*“Before this course*,* I did not know how prehospital collaboration between the police*,* ambulance services*,* and emergency service worked. It has been eye-opening to see how responsibilities are shared before a patient even reaches the hospital.”*

The program also illuminated the logistical and communication challenges inherent in interdepartmental and cross-sectoral collaborations in the hospital. For instance, participants’ emphasized the importance of “breaking down silos” within the hospital:

*“We need more channels for planning and communication across departments…”.*

*“We often operate in isolation within our specialties*,* this course helped me realize how crucial it is to develop streamlined communication structures and unified approaches*,* especially when facing extraordinary inflows of patients. Understanding how much we rely on each other in such situations has been transformative"​.*

#### Organizational structures

Beyond individual learning, the program had tangible impacts at the organizational level within the hospital. Several participants later became preparedness coordinators within their departments after the program, highlighting the educational role in capacity-building for the hospital’s disaster response framework. The program also tried to promote a sense of professional growth among participants’, many of whom expressed a newfound interest in pursuing careers in disaster medicine. One participant reflected:

*“This course has given me a strong foundation to shape my role in disaster preparedness within my department.”*

#### Suggestions for improvements of the program

Some feedback from the participants on the quality and structure of the program provided insights into possible areas for improvement. There were recommendations for a more structured progression of program topics, moving from local and regional issues to national and international perspectives rather than the opposite, i.e., how this program started. One participant suggested:

*“Starting with local contexts would have better prepared us for the later discussions on international humanitarian law and global disaster management.”*

There was also a call for greater involvement of hospital first-line managers and leadership in the program to ensure alignment between participant learnings and organizational goals. Furthermore, participants’ advocated for a more explicit integration of security considerations. They emphasized collaboration with law enforcement and emergency services could be further developed beyond the current program curriculum.

## Discussion

Earlier studies highlight the need for standardized, competency-based disaster management education. European and global frameworks aim to bridge training gaps, promote interdisciplinary collaboration, and utilize technology to equip disaster managers for contemporary challenges [1–3, 5–7]. This study aimed to evaluate the outcome of one such initiative, the newly established PRAD-MED program in disaster medicine training at Sahlgrenska University Hospital in Sweden.

The results reinforce the value of structured education for healthcare professionals, suggesting the benefits of integrating leadership training, cross-sector collaboration, and practical exercises such as 3LC and MACSIM simulations [13–15]. The program demonstrated potential in improving knowledge, confidence, and preparedness for managing disaster scenarios.

### Improved knowledge and confidence

Quantitative results indicate a significant improvement in self-reported knowledge of disaster medicine, with 94% of participants’ rating their post-program knowledge as “high” or “very high.” The emphasis on leadership, communication, and interdisciplinary coordination appears to promote the skills required to manage complex crisis situations. Qualitative feedback supported these findings, with participants’ frequently highlighting the hands-on simulation exercises as key for building confidence and readiness for leadership roles in disaster contexts. These results are consistent with previous studies suggesting that increased knowledge is closely linked to improved confidence and performance in complex emergency environments [30–32]. Although the quantitative component relied on self-reported improvement, complementary data from CSCATTT-based observations, structured peer feedback, and end-of-course group interviews corroborated the findings, demonstrating alignment with mixed-methods educational frameworks in disaster medicine [18–21]. The continued engagement of participants’ as certified MACSIM simulation instructors and departmental preparedness coordinators further indicate that the observed effects extended beyond perception toward sustained organizational impact.

### Leadership competence in crisis management

The findings indicate that the program helped cultivate leadership competence integrating routine managerial effectiveness with the flexibility and decisiveness required during disasters. This outcome was likely achieved through two main mechanisms:

1. The program continuously emphasized practical leadership using the CSCATTT model, with “C” representing command and control, highlighting both vertical and horizontal leadership and collaboration with relevant stakeholders. For many participants, this represented a new dimension of leadership training extending beyond clinical supervision to encompass organizational and interagency command.
2. The program also emphasized decision-making under pressure, highlighting the importance of assuming accountability for critical decisions in dynamic and uncertain situations, in contrast to the consensus-based processes common in everyday clinical practice.

Furthermore, participants identified challenges in adapting traditional hospital hierarchies to dynamic disaster scenarios and emphasized that the MACSIM exercises provided valuable insight into managing mass-casualty incidents. These hands-on simulations underscored the importance of adaptability, clear communication, and coordinated teamwork, which are competencies often underrepresented in conventional medical curricula. Similar patterns have been reported in previous research on the management of mass-casualty incidents and in structured simulation-based training programs for major incident management [16, 33–35].

### Interdisciplinary and cross-sectoral collaboration

Participants’ reported enhanced understanding of the roles of multiple stakeholders in disaster settings, including prehospital services, law enforcement, and government agencies. The program appeared to promote appreciation for interdepartmental and cross-sectoral communication, with participants’ frequently highlighting the importance of improving coordination across hospital departments. This awareness of shared responsibilities and interdependent workflows is recognized as a crucial factor in effective disaster response, as documented by other researchers [36–39].

Balancing local, regional, national, and international perspectives emerged as an important point of reflection. While all dimensions were valuable, focusing on strategic rather than purely operational aspects appeared essential to maintain coherence within the program. Standardizing core content may further enhance quality and continuity, with careful selection of international components that align with the program’s overall objectives [1–3, 5–7].

A significant outcome was the collaboration with the hospital’s safety and preparedness unit. This partnership facilitated the integration of training within the institution’s core preparedness processes, helping ensure that acquired knowledge and skills became a long-term organizational asset. Strengthening the dissemination of knowledge throughout the hospital, from executive leadership to individual clinical departments, was identified as a key next step [39, 40].

Finally, improving collaboration both within and beyond hospital systems was recognized as a major opportunity. Strengthening partnerships with regional operational centers, municipal authorities, and emergency services appeared to enhance overall preparedness and may contribute to the development of a broader, more resilient disaster response network [41–43]. Further involvement of these external actors in future program iterations could support cross-sectoral exercises and foster strategic partnerships, enhancing resilience and preparedness at regional and national levels.

### Organizational impact and professional growth

Beyond individual learning, the program contributed to institutional capacity-building by encouraging participants to take active leadership roles in disaster preparedness. Many participants subsequently assumed positions as preparedness coordinators within their departments, demonstrating success in fostering long-term professional growth and institutional engagement [1–3, 7]. This highlights the potential of structured disaster medicine education to influence organizational preparedness frameworks and strengthen institutional response capacity which has previously been identified as central by researchers [4].

### Future directions and suggested improvements

Building on these outcomes, several lessons emerged to guide the program’s continued development. A notable aspect was the involvement of hospital management and the participants’ immediate supervisors in the training process. Their engagement appeared to support departmental initiatives and may have increased the likelihood of sustained organizational impact. However, an important improvement would be to involve these managers earlier in the course. In the present cohort, they were invited only at a late stage to attend a session featuring presentations of participants’ departmental preparedness assignments. Early engagement would allow for management-level discussions and better utilization of the participants’ expertise to drive strategic improvements and practical changes in day-to-day preparedness efforts.

Future iterations of the program could also adopt a more structured progression from local to global perspectives to enhance coherence in disaster management education. Broader inclusion of stakeholders, such as hospital administration, regional health authorities, and external emergency services would further align the program with organizational goals and strengthen interagency collaboration. Additionally, a deeper focus on security and intersectoral coordination is needed. Incorporating advanced technologies such as augmented and virtual reality could enrich learning experiences, particularly in settings with limited access to physical training resources [44].

A central focus of the PRAD-MED program has been the education of future instructors in disaster medicine and simulation-based training at local, regional, and national levels. This initiative has already resulted in refresher meetings, subsequent course iterations, and ongoing alumni activities that combine social networking, peer support, and regular follow-up meetings to report departmental progress. These mechanisms have facilitated the continuous improvement of contingency plans and contributed to sustainability across the hospital’s preparedness system.

## Limitations

The study has several limitations that should be considered. First, the sample size was small, involving one cohort of 16 participants from a single institution. Although the results can be transferred to comparable contexts, they are not statistically generalizable to other settings or professional groups.

Second, data were collected immediately after program completion. While this ensured a high response rate and rich feedback, it primarily captured short-term impressions. To strengthen long-term retention and practical application, the program incorporated instructor-led activities and alumni follow-up sessions in which participants reported on ongoing preparedness tasks and the progression of their departments’ disaster readiness. These continued activities, along with the participants’ roles as preparedness coordinators and certified disaster simulation instructors, provided an early indication of institutional learning and sustained competence development.

Third, although established evaluation models such as Kirkpatrick’s could have been applied, the outcome evaluation in this study relied mainly on self-assessed improvement, which may not directly reflect objectively measured competence or performance. Self-assessment supports reflective learning and helps identify perceived strengths and weaknesses, but it is inherently subjective. To mitigate this limitation, the program incorporated structured and triangulated evaluation tools, including CSCATTT-based observational assessment, peer and instructor feedback, and a final focus group interview, in line with recommended mixed-methods practices [24–26].

A further limitation concerns the qualitative group interview format, which involved all 16 participants simultaneously. While this approach allowed for broad input and group-level reflection, it risked unequal participation and social desirability bias. These risks were mitigated by experienced moderators using structured prompts, defined objectives, and independent scorecards. The interviewers also conducted a debrief session after the interview to calibrate evaluations and improve consistency [45, 46].

Regarding the qualitative analysis, findings were derived through systematic inductive content analysis but presented via selected quotations rather than exhaustive thematic tables. While this limits the visibility of the full range of responses, it aligns with established qualitative reporting practices that emphasize illustrative depth and clarity over enumeration [47].

Finally, the course was based on an American disaster medicine curriculum delivered in English, which is widely used internationally. Nevertheless, other curricula developed in different linguistic or regional contexts may yield different experiences and outcomes.

Future studies should include multi-cohort designs, delayed follow-up assessments, and objective performance evaluations to better quantify long-term retention, behavioral change, and the broader impact on disaster preparedness and response across healthcare systems.

## Conclusions

In conclusion, the PRAD-MED program aimed to address important gaps in disaster management training and may contribute to strengthening both individual competencies and institutional preparedness. Its integrated design, combining theoretical instruction with collaborative simulation exercises, seems to support improvements in leadership, communication, and coordination skills while establishing a framework for continued education.

Early outcomes, such as appointing participants as preparedness coordinators, certified instructors, and creating a structured exercise platform, suggest potential organizational benefits; however, these findings are based on short-term observations and require further validation.

Future steps, including curriculum refinement, broader stakeholder engagement, and inclusion of diverse healthcare roles, could enhance the program’s reach and sustainability. With additional development, such as incorporating research components, the program could evolve into a postgraduate qualification, fostering long-term competence development and contributing to national preparedness efforts. Nonetheless, ongoing evaluation will be essential to confirm these impacts over time.

## Supplementary Information

Supplementary Material 1. Appendix

## Data Availability

All available data and materials are included in the methodology and results section. Any other information can be provided by corresponding author upon reasonable request.

## Abbreviations

PRAD-MED: Preparedness and Disaster Medicine
CSCATTT: Command and Control, Safety, Communication, Assessment, Triage, Treatment, and Transport
MRMI: Medical Response to Major Incidents
MACSIM: Mass Casualty SIMulation
MIMMS: Major Incident Medical Management and Support
Three-Level Collaboration: 3LC
