# Supplementary material for: Proactive postgraduate education in disaster medicine and preparedness for enhanced disaster management
Source: BMC Med Educ. 2026 Feb 14;26:336. doi: 10.1186/s12909-026-08638-5 (PMC12930613; doi:10.1186/s12909-026-08638-5)
Supplement: Supplementary file 1 — Supplementary Material 1. Appendix [file 12909_2026_8638_MOESM1_ESM.docx]

Appendix

Table 1:

| Domains | Definition | Method | Content |
| --- | --- | --- | --- |
| **Identification of Core Learning Domains** | "Core learning domains refer to the foundational subject matter areas that outline the essential knowledge required for disaster medicine and management. These domains serve as the theoretical bedrock for performance." | "Initial identification of these domains involved analysis of existing disaster medicine curricula, review of international guidelines, and expert interviews with seasoned disaster responders. We synthesized common themes and established three core learning domains, mirroring the structure of the provided modules | - Disaster Medicine Organization, Leadership, and Governance: This domain encompasses the foundational knowledge related to the structural, policy, and legal aspects of disaster response, both nationally and internationally. It includes understanding crisis organizations, global humanitarian systems, international law, civil-military collaboration, and leadership principles in emergencies. - Disaster Medicine Knowledge, Skills, and Training: This domain focuses on the theoretical and practical knowledge required for tactical application in disaster settings. It includes principles of disaster triage, medical decision-making under pressure, and understanding simulation methodologies. - Disaster Medicine Practice, Training, and Collaboration: This domain emphasizes the knowledge required for effective multi-stakeholder collaboration and practical application in large-scale disaster scenarios, including cross-sectoral planning and advanced exercise methodologies" [1-4]. |
| **Delineation of Competency Domains** | "Competency domains represent overarching clusters of related skills, behaviors, and attributes, emphasizing how knowledge is applied in practice through observable actions. They move beyond factual recall to focus on applied ability." | "Following the identification of core learning domains, we engaged in **functional analysis of critical disaster response roles and conducted workshops with key stakeholders (e.g., emergency medical services, military, government agencies)** to delineate the key areas of performance. This resulted in **three** competency domains: | - **Foundations and Policy (Aligned with Module 1):** This domain groups competencies related to understanding and navigating the organizational, legal, and policy landscapes of disaster response. It includes abilities such as understanding the Swedish total defense system, navigating crisis organization structures, applying international humanitarian law, and demonstrating leadership in command systems. - **Tactical Application (Aligned with Module 2):** This domain encompasses competencies related to the practical and immediate application of medical knowledge and decision-making in a disaster environment. It includes skills such as performing disaster triage effectively, making sound medical decisions under extreme pressure, and utilizing simulation tools for training and assessment. - **Collaborative Exercises (Aligned with Module 3):** This domain focuses on competencies essential for effective inter-agency and inter-professional collaboration during large-scale disaster responses. It includes abilities such as coordinating with diverse stakeholders, engaging in cross-sectoral planning, and participating in and facilitating complex multi-agency exercises" [5-6]. |
| **Construction of the Competency Framework** | "The competency framework is the comprehensive, structured model that integrates all identified competencies, organized within their respective competency domains, to provide a holistic blueprint for successful performance. It specifies the expected behaviors and often different levels of proficiency." | "The identified core learning domains and competency domains formed the foundation for constructing the **Disaster Medicine Readiness Competency Framework**. | For each competency domain, we developed specific competencies, each detailed with **observable behavioral indicators and, where applicable, different proficiency levels (e.g., novice, proficient, expert)**. This iterative process involved **validation surveys with experienced disaster medicine practitioners and expert panel reviews to ensure relevance and comprehensiveness**. The complete framework provides **a robust guide for curriculum development, a standardized tool for performance assessment, and a clear basis for ongoing professional development and training initiatives in disaster medicine**" [7-9]. |

**References**

1. **Walsh, L., Subbarao, I., Gebbie, K., et al. (2012). Core Competencies for Disaster Medicine and Public Health. *Disaster Medicine and Public Health Preparedness, 6*(1), 44-52.** This is a foundational paper that outlines a consensus-based set of core competencies for disaster medicine and public health, developed by a broad expert working group. It is highly relevant for defining core learning domains and competency domains.
2. **National Academies of Sciences, Engineering, and Medicine; Health and Medicine Division; Board on Population Health and Public Health Practice; Board on Health Sciences Policy; Committee on Evidence-Based Practices for Public Health Emergency Preparedness and Response. (2020). *Evidence-Based Practice for Public Health Emergency Preparedness and Response*. National Academies Press (US).** This publication offers a comprehensive overview of evidence-based practices in public health emergency preparedness and response, implicitly supporting the need for defined competencies.
3. **Ablah, E., Weist, E. M., McElligott, J. E., et al. (2013). Public health preparedness and response competency model methodology. *American Journal of Disaster Medicine, 8*(1), 49–56.** This article discusses methodologies used in developing public health preparedness and response competency models, providing insights into the "Method" sections of your framework.
4. **World Health Organization. (2022). *Global Competency and Outcomes Framework for Universal Health Coverage*. Geneva: World Health Organization.** While broader than just disaster medicine, this framework provides a robust model for competency-based education in health and includes emergency preparedness and response as key areas, offering valuable insights into framework construction.
5. **Powell, R. A., & Single, H. M. (1996). Focus groups. *International Journal for Quality in Health Care, 8*(5), 499–504.** This reference supports the use of focus groups (a type of expert interview/stakeholder workshop) as a method for qualitative data collection in defining competencies.
6. **Daily, S. S., Padjen, J. A., & Birnbaum, M. L. (2020). Nontechnical Competency Framework for Health Professionals in All-Hazard Emergency Environment: A Systematic Review. *Disaster Medicine and Public Health Preparedness, 14*(1), 1-10.** This systematic review highlights various methods used to develop non-technical competency frameworks for health professionals, including those in emergency environments, supporting the methodological approaches you've outlined.
7. **Rashid, H., Alexakis, L. C., & Pereira, I. (2025). Disaster Medicine Education for Medical Students: A Scoping Review. *Cureus, 16*(12), e75035.** (Note: This is listed as a future publication in some databases, so check for the final publication date if citing formally). This recent scoping review provides a comprehensive overview of the literature on disaster medicine education for medical students, emphasizing the need for structured curricula and competency development.
8. **Byrne, D., Ashcroft, R., & Alexander, M. (2024). Evaluation of a Disaster Preparedness Curriculum and Medical Students’ Views on Preparedness Education Requirements for Health Professionals. *Disaster Medicine and Public Health Preparedness*.** This article directly addresses the evaluation of disaster preparedness curricula and the perceived needs for training among medical students, underscoring the importance of your framework.
9. **Ersoz, M. A., & Gultekin, H. T. (2025). The impact of a disaster medicine clinical training program on medical students' disaster literacy. *PeerJ, 13*, e18800.** This research demonstrates the effectiveness of disaster medicine training programs in improving disaster literacy and response skills, reinforcing the "desired outcome" of your framework.
